# No Difference in Sleep and RBD between Different Types of Patients with Multiple System Atrophy: A Pilot Video-Polysomnographical Study

**DOI:** 10.1155/2013/258390

**Published:** 2013-03-28

**Authors:** Maria-Lucia Muntean, Friederike Sixel-Döring, Claudia Trenkwalder

**Affiliations:** ^1^Paracelsus Elena Klinik, Klinik Straße 16, 34128 Kassel, Germany; ^2^Department of Neurosciences, University of Medicine and Pharmacy, Victor Babes Straße 43, 400012 Cluj-Napoca, Romania; ^3^Department of Neurosurgery, University of Göttingen, Robert Koch Straße 40, 37075 Göttingen, Germany

## Abstract

*Background*. Patients with multiple system atrophy (MSA), similarly to patients with alpha-synucleinopathies, can present with different sleep problems. We sought to analyze sleep problems in the two subtypes of the disease MSA cerebellar type (MSA-C) and MSA parkinsonian type (MSA-P), paying special attention to REM sleep disturbances and periodic limb movements (PLMs). *Methods*. In the study we included 11 MSA-C and 27 MSA-P patients who underwent one night polysomnography. For the analysis, there were 37 valid polysomnographic studies. *Results*. Sleep efficiency was decreased in both groups (MSA-C, 64.27% ± 12.04%; MSA-P, 60.64% ± 6.01%). The PLM indices using standard measures, in sleep (PLMS) and while awake (PLMW), were high in both groups (MSA-C patients: PLMS index 72 ± 65, PLMW index 38 ± 33; MSA-P patients: PLMS index 66 ± 63, PLMW index 48 ± 37). Almost one-third of the MSA patients of both groups presented features of RLS on video-polysomnography. RBD was described in 8/11 (73%) patients with MSA-C and 19/25 (76%) patients with MSA-P (*P* = 0.849). *Conclusion*. Our results showed very similar polysomnographic results for both MSA-P and MSA-C patients as a probable indicator for the similar pathologic mechanism of the disease and especially of its sleep problems.

## 1. Introduction

Nighttime sleep disturbances are a recognized problem in multiple system atrophy (MSA) patients. These disturbances have long been observed, but the studies published to date have only included small sample sizes. Nighttime sleep problems in patients with MSA include REM sleep behavior disorder (RBD) [1], periodic limb movements (PLMs) [2], restless legs syndrome (RLS) [2], or RLS like symptoms [3–6]. All of them lead to sleep fragmentation and decreased sleep efficiency [7, 8].

In MSA, as in other alpha-synucleinopathies, RBD can be present, with a prevalence of up to 90% [9], and sometimes can antedate the occurrence of motor symptoms [10, 11]. RBD and REM sleep without atonia (RWA) in MSA and PD patients may be related to lesions of brainstem nuclei and pontomedullary pathways, as suggested in previous studies [3, 12].

On the other hand, clinically defined RLS seems to be less frequent in MSA patients when compared to patients with other synucleinopathies [13]. However, most patients with RLS have PLMs during sleep, an unspecific sign of both RLS or other sleep disorders. The pathology of PLMs in patients with parkinsonism is not yet clarified, but a few theories have been put forward. One hypothesis concerns the involvement of the dopaminergic system of neurotransmission [14]. A second hypothesis is the suprasegmental disinhibition at the brainstem or spinal cord level [15].

There is only sparse data in the published literature comparing the polysomnographic (PSG) parameters in MSA patients and referring to the two different types of the disease according to Gilman et al.'s classification [16]. In this context, we aimed to analyze sleep problems in the two subtypes of MSA patients (MSA-C and MSA-P) and paid special attention to REM sleep disturbances and limb movements during the night.

## 2. Methods

The study was approved by the Ethics Committee of the Landesärztekammer Hessen in the context of Parkinson Syndromes in PSG studies. All subjects agreed to take part in the study and signed a written informed consent form that included the video-assessment.

We enrolled 38 patients with both MSA-P and MSA-C in this sleep laboratory study. The patients were referred for diagnosis or treatment of their disease and were investigated in our sleep lab because of subjective nighttime sleep problems, RLS like symptoms, probable RBD, or other nighttime disturbances including suspicious stridor. Three patients were evaluated as *de novo* Parkinson syndromes, who later proved to be MSA patients. 

The demographical data, clinical findings, concomitant diseases, medication during the day, when the polysomnography was performed and Parkinson Disease Sleep Scale-version 2, (PDSS-2) scores [18], when available, were obtained. We paid special attention to comorbidities such as cardiovascular and psychiatric conditions. The data were obtained from the admission charts of the patients who were previously diagnosed by cardiologists or psychiatrists. 

### 2.1. Diagnosis

The diagnosis of MSA was established according to the 2008 Consensus Criteria established by Gilman et al. [16]. Patients were separated into two groups: MSA with predominant parkinsonism (MSA-P) and MSA with predominant cerebellar ataxia (MSA-C). Clinical symptoms of patients are described in Table 1.

### 2.2. Polysomnography

Nighttime sleep recordings started immediately after connecting the patient and calibration with lights off at 22:00 and ended at 6:00 the next morning. Cardiorespiratory PSG (Xltec: Excel Tech Ltd., Oakville, ON, Canada) was applied including bilateral monopolar central electroencephalography (EEG) with two channels, electrooculogram (EOG), chin and bilateral tibialis anterior surface electromyography (EMG), air flow registration, tracheal sound registration by microphone, thoracic and abdominal belts to measure respiratory movements, electrocardiography (EKG), and oximetry. All patients were documented with an infrared video recording synchronized to the PSG. A sleep laboratory technician monitored each recording. Sleep (including sleep stages), PLMs, and apneas were scored visually by a trained technician according to standard criteria [1, 19]. PLMs were scored in sleep and in wakefulness only if they occurred in a series of at least four consecutive movements lasting 0.5 to 5 seconds each with an intermovement interval of 4 to 90 seconds, in accordance with international scoring rules [2, 20]. The number of PLMs per hour of time in bed (PLM index), and the number of PLMs during wakefulness per hour of wake time (PLMW index), the number of PLMs during sleep per hour of total sleep time (PLMS index) were evaluated separately. All sleep evaluations were reviewed and supervised by board-certified sleep specialists. Sleep efficiency was defined as total sleep time (TST)/time in bed (TIB). Quantitative analysis of sleep stages was calculated as a percentage of TST. RBD was diagnosed by second per second review in time-synchronized video analysis of all REM episodes by experienced raters in accordance with EEG, EOG, and chin EMG. RBD was defined as the presence of REM sleep without atonia (RWA) together with complex movements or vocalizations during REM sleep apparently associated with dreaming or dream-enacting behaviors visible in time-synchronized video-PSG according to criteria established by Schenck et al. [21] and the International Classification of Sleep Disorders, second edition (ICSD-2) [1] with one modification as historical information was not included. Severity of RBD was quantified using the RBD severity scale (RBDSS). On the RBDSS, motor events in REM sleep were rated on a digital scale from 0–3 according to the localization and severity of movements. The scales rates the following: no visible movement but registration of RWA scored as 0, slight movements including facial movements, jerks or movements restricted to the distal extremities scored as 1, movements involving the proximal extremities, complex and/or violent behaviors scored as 2, and any axial involvement with a possibility of falling or observed falls scored as 3; vocalizations were rated as absent, indicated by “0”, or present, indicated by “1”, for any sound generated during REM sleep other than respiratory noises. Motor and vocalization scores were separated by a full stop [22]. 

We analysed RWA using chin EMG activity in REM sleep according to the criteria published by Frauscher et al. [23]. We evaluated the mentalis muscle. For scoring phasic or tonic EMG activity, the recording was divided into 3 sec mini-epochs. Each 3 sec mini-epoch was scored as having or not having “any” EMG activity, irrespective of whether it contained tonic, phasic, or a combination of both EMG activities. Finally, we also calculated the percentage of 3 sec mini-epochs with “any” chin EMG activity.

RLS-like symptoms were defined according to the video-polysomnographic assessment and clinical interview after the sleep study in those subjects, when PLMS and restlessness were obvious, since there is no structured targeted interview for RLS patients with Parkinson syndromes available. If not all 4 diagnostic features were fulfilled or could be obtained, we called it RLS-like syndrome.

Sleep apneas were defined as an apnea-hypnea index (AHI) of 5 or more in accordance with Ruehland et al. [24].

### 2.3. Analysis

The statistical analysis was made in an environment for statistical computing and graphics, (“R”, version 1.15.1) [25]. The association between two qualitative variables was assessed using the Fisher exact test. The strength of the association was assessed with odds ratio along with the 95% confidence intervals. To check for differences between two independent groups of quantitative data, Mann Whitney *U* test was used.

## 3. Results

### 3.1. Patient Population

**Figure 1:**
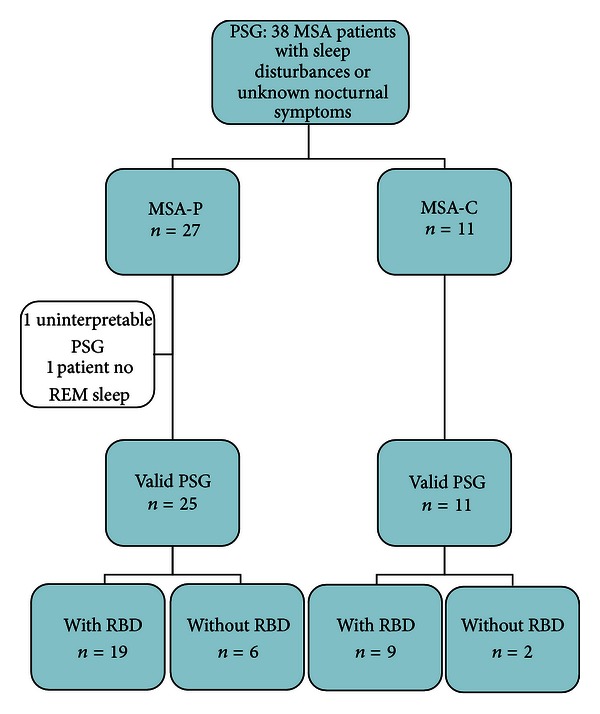
Study tree of the MSA patients. RBD = REM sleep behavior disorder, PSG = polysomnography.

Thirty-eight patients with MSA were referred to the sleep laboratory within approximately 2 years, out of a total of 50 MSA patients who were hospitalized in the Paracelsus Elena-Klinik at that time. One patient with MSA-P had a sleep efficiency of only 4% in the PSG, thus providing insufficient sleep to allow further analysis. This patient was excluded from the study. Therefore the study group consisted of 37 MSA patients with interpretable PGSs. There were 19 females and 18 males with an average age of 66.62 ± 8.21 years. The study tree of patients is presented in Figure 1. The MSA-C group consisted of 11 patients (5 females and 6 males, average age 67.64 ± 5.55) and the MSA-P group consisted of 26 patients (14 females and 10 males, average age 66.19 ± 9.17). One patient from the MSA-P group failed to enter REM sleep; therefore this patient was excluded from the REM sleep analysis. The demographics of the study population, together with the concurrent medical conditions and medication, are presented in Table 1.

### 3.2. Medical Conditions

The body mass index (BMI) did not differ significantly between the two groups. The incidences of cardiovascular and psychiatric comorbidities in MSA-C and MSA-P patients were not significantly different. 

Daily levodopa dose was higher in patients with MSA-P compared to MSA-C patients (*P* = 0.017). No difference was observed in the dosage and number of patients using dopamine agonists, amantadine, selective serotonin reuptake inhibitors (SSRIs), and opioids in the two MSA patient groups.

### 3.3. Subjective Sleep Evaluation

The overall subjective quality of nighttime sleep (item 1 score on PDSS-2) was 1.55 ± 1.08 for MSA-C patients and 1.59 ± 1.23 for MSA-P patients, with no statistically significant difference. The total PDSS-2 scores were as follows: 17.91 ± 4.41 in the MSA-C group and 19.64 ± 6.90 in the MSA-P group (no statistically significant difference).

### 3.4. Sleep Parameters

**Figure 2:**
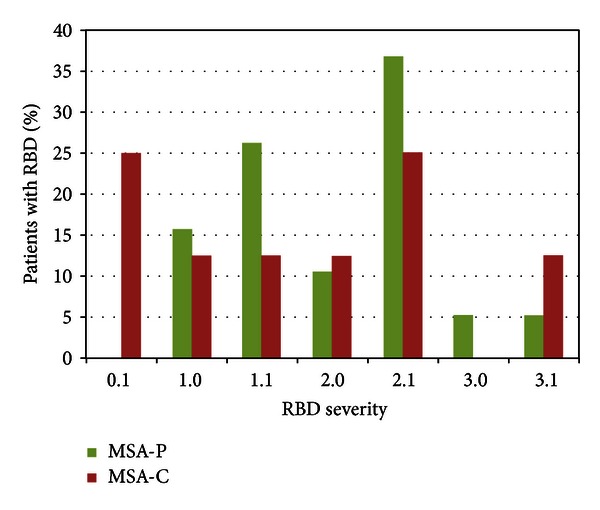
Representation of MSA patients according to RBD severity evaluated with RBDSS. RBSS = REM sleep behavior disorder severity scale, MSA-C = multiple system atrophy cerebellar predominant, MSA-P = multiple system atrophy parkinsonian predominant.

The results of the PSG studies of the two subtypes of MSA patients are shown in Table 2 and results of RWA in both subtypes are shown in Table 3. RBD (according to the modified ICSD-2 criteria [1]) was described in 8/11 (73%) patients with MSA-C and 19/25 (76%) patients with MSA-P. There was no difference in the incidence as well as in the severity of clinical RBD between the two groups (*P* = 0.849). Violent manifestations were noted in only two of the MSA-P and one of the MSA-C patients. Details of RBD severity are represented in Figure 2.

The results of the EMG analysis of the mentalis muscle in relation to RBD are presented in Table 3. These show that MSA-C patients with RBD presented an average percentage of EMG activity in REM sleep of 46.79% ± 21.17% < compared to an average percentage of only 7.35% ± 7.94% for patients without RBD. In the MSA-P group the average percentage of EMG activity for patients with RBD was 51% ± 36.96% compared to only 17.72% ± 31.51% for the patients without RBD. The comparison between the RWA in both groups with RBD was not significantly different.

In the MSA-C group, 3 patients with RBD were using SSRIs, and none from the patients without RBD. In the MSA-P group, 8 patients with RBD were using SSRIs, compared to 3 patients without RBD. No statistical analysis was performed, due to the small number of patients in each group.

Respiratory events of MSA patients are represented in Table 4.

## 4. Discussion

Our study population consisted of MSA patients who were referred for sleep studies due to different or unclear nighttime sleep disturbances. The PSG results of the two groups of MSA patients (MSA-C and MSA-C) could be compared due to similarities in patient demographics and in clinical features. 

We noted a high frequency of sleep problems in MSA patients both MSA-C and MSA-P, consistent with the results of Wetter et al. and Tison et al. [3, 11]. Taking into account the localization of the pathologic processes in the two MSA subtypes we expected to find different nighttime sleep problems in the two groups. The results of the study, however, which showed very similar nighttime problems and PSG results for both MSA-P and MSA-C patients, are a probable indicator for the similar pathologic mechanism of the disease and especially of its sleep problems. This is mainly a pilot study with a descriptive analysis and due to the small number of participants in each subgroup statistically significant differences cannot be expected. It could be considered a preliminary study, and more patients should be investigated to confirm that there is no difference in sleep parameters of these two subgroups of MSA patients.

The subjective sleep evaluation was undertaken using the PDSS-2 scale. Overall quality of sleep assessed by the first question of the scale showed a moderate quality of sleep. No difference was observed between the two MSA subtype patient groups concerning subjective nighttime sleep problems.

Sleep efficiency was decreased in both MSA groups (64.27% ± 12.04%, resp. 60.64% ± 16.01%) compared to the values of elderly normals available in the literature (76.5% ± 2.2%) [26]. MSA patients also presented fragmentation of sleep during the night either due to limb movements or to sleep apnea.

The assessment of motor activity in sleep for the patients included in the study was performed using the standard criteria for routine practice in our sleep laboratory at that time. Some authors use more quantitative data to analyze motor parameters in sleep, for example, using the periodicity index [27]. We consider it an important new method, but it is, until now, not the gold standard for analysing PLMs in neurodegeneration. 

An important finding of our study is the fact that MSA patients in the sleep laboratory featured a high number of PLMs. The PLM indices, both PLM during sleep and PLM while awake, were much higher (MSA-C patients: PLM index 64 ± 55; MSA-P patients: PLM index 61 ± 48) than those reported in PD patients in the same PSG laboratory (PLM index 36 ± 40) [28]. Iranzo et al. found a similarly high index of PLMs in MSA patients, also using standard PLM assessments, which could suggest a dysfunction in the structures that modulate sleep [29]. One explanation for the difference between PLM indices between MSA and PD patients could be that the spinal-cerebellar pathways involved in PLM generation are probably more affected in MSA than in PD patients. It would therefore be of interest to use more elaborated assessments for measuring PLMS, such as the periodicity index [27] to see, if the difference between PD, RLS, and MSA is related to movements with periodicity or just to single movements while motor activity is increased during sleep. The overall dosages of dopamine agonists in MSA patients were in general lower compared to PD patients, which could also contribute to an increased number of limb movements in MSA patients.

Almost a third of the MSA patients presented RLS features on the video-PSG with obvious restlessness at night in the PSG-video. Spontaneous reporting of these symptoms, however, is rare. This implies that a more thorough questioning of MSA patients about RLS is needed in order to detect these symptoms before referring patients to sleep studies. We classified these patients as “RLS-like” syndrome as used by others [5] when not all diagnostic features were met or obtained.

We assumed that due to the more specific involvement of pontine and cerebellar structures including the olivoponto-cerebellar structures in the pathology of MSA-P [30], these patients should have a higher prevalence of RLS than MSA-C patients. The results of the study, however, did not support this hypothesis. 

The presence of RWA was high in both MSA patient groups. We analysed the chin EMG activity in sleep with the method described by Frauscher et al. [23] although the method has not been validated for MSA patients. Thus we noted a high percentage of EMG activity in MSA patients with RBD compared to MSA patients without RBD in both MSA subtypes (for MSA-C patients 46,79% versus 7,35% and for MSA-P patients 51% versus 17,72%). There was an interindividual variability of chin EMG activity, but the mean values fall within the range given by Frauscher et al.. The cut-off value for 100% specificity for RBD in PD patients was 18,2% in the group of Frauscher et al. [23]. This comparison deserves attention, as it is the first study that uses the Frauscher method in MSA patients.

Our results are supported by the work of Nomura et al. who revealed RWA in 68.8% of patients with MSA [31]. 

The relationship between SSRIs and the presence of polysomnographically-diagnosed RBD could not be statistically analysed due to the small number of patients in each group. In the literature, Frauscher et al. showed a significant association between RBD and SSRI use in a population with different sleep disorders, but not in neurodegenerative patients [32].

SSRIs were used in therapeutic doses either for depressive symptoms or with the aim of improving MSA symptoms. It is assumed that a serotonergic depletion occurs in the brainstem nuclei of MSA-patients [33]. A first small randomized trial of our group could show improvement of motor symptoms and speech with paroxetine in MSA patients [34]. In our current study, more MSA-P patients than MSA-C patients have been treated with SSRIs, although the difference between the two groups did not reach the significance threshold. The neurodegenerative changes of the brain or neuropharmacological alterations leading to RBD may therefore be more important than any pharmacological imbalance that is caused by serotonergic treatment in patients, who are not or not yet diagnosed with neurodegeneration. 

A number of MSA patients in both groups presented with sleep apnea that could be explained either by the pathologic process of the disease or by an increase in BMI. The average values in both groups were situated above the normal weight limit. The upper normal weight limit according to the World Health Organization is 24.99 kg/m^2^. This shows that many of the MSA patients were overweight, which could be a contributing factor to their nighttime sleep disturbances, especially their respiratory problems [35]. However, PD patients have similar BMIs, and it is still unclear if neurodegeneration leads to preponderance because of immobility or if those who are overweight have a higher risk of parkinsonism [36].

This study showed once more that sleep problems in MSA patients represent a frequent and serious problem. Patients are referred to the sleep laboratory for various nocturnal complaints, which were difficult to diagnose from history only. After PSG analysis their subjective sleep disturbances proved to be sleep fragmentation, RBD, PLMS, RLS, or several phenomena combined. One of the limitations of this study is represented by the fact that we included only MSA patients who had subjective complaints of sleep, and not the entire group of MSA patients.

Even though the pathology of the two subtypes of MSA is somewhat different we did not notice any significant difference in the occurrence of sleep disturbances in MSA-C and MSA-P patients thus possibly arguing for a common disease entity. This could be considered as a preliminary study. More patients are expected to be included in this type of analysis in order to better clarify the results.

## Figures and Tables

**Table 1:** Description of the study population with valid PSGs.

Demographics	MSA-C (*n* = 11) (29.73%)	MSA-P (*n* = 26) (70.27%)	*P* value
Female (%)	5 (45.45)	14 (54.55)	NS
Male (%)	6 (53.85)	12 (46.15)	NS
Age, years	67.64 ± 5.55	66.19 ± 9.17	NS
Disease duration, years	4.27 ± 2.76	3.44 ± 2.28	NS
BMI	27.28 ± 3.07	27.61 ± 4.50	NS
MMSE	26.36 ± 4.82	28 ± 1.37	NS
Signs and symptoms, *n* (%)			
Cerebellar	11 (100)	3 (11.54)	0.000
Extrapyramidal	10 (90.91)	26 (100)	NS
Pyramidal	2 (18.18)	6 (23.08)	NS
Concomitant diseases, *n* (%)			
Cardiovascular*	4 (36.36)	12 (46.15)	NS
Psychiatric**	3 (27.27)	9 (34.61)	NS
Medication			
L-dopa, mg/day	391.67 ± 270.49	747.89 ± 439.50	0.017
*n* (%)	6 (55)	19 (73)	NS
Dopamine agonists, mg/day	0	319.67 ± 299.47	NA
*n* (%)	0	6 (23)	NA
Amantadine, mg/day	300	250 ± 100	NS
*n* (%)	1 (9.09)	4 (15.38)	NS
SSRIs, *n* (%)	3 (27.27)	11 (42.31)	NS
Opioids, *n* (%)	1 (9.09)	1 (3.85)	NS
Benzodiazepines, *n* (%)	2 (18.18)	6 (23.08)	NS
Other antipsychotics, *n* (%)	2 (18.18)	0	NA

Values are mean ± SD, Dopamine agonists dose = L-dopa equivalent dose according to Tomlinson et al., 2010 [17], MSA-C: multiple system atrophy cerebellar predominant, MSA-P: multiple system atrophy parkinsonian predominant, BMI: body mass index, MMSE: Mini Mental State Examination, L-dopa: levodopa, SSRIs: selective serotonin reuptake inhibitors, NS: not significant (*P* value > 0.05), NA: not applicable.

*Clinically relevant hypertension or any other form of heart disease. **Clinically relevant psychiatric conditions.

**Table 2:** Polysomnographic characteristics of MSA-C and MSA-P patients.

	MSA-C (*n* = 11)	MSA-P (*n* = 26)	Significance *P* value
Sleep efficiency, %	64.27 ± 12.04	60.64 ± 16.01	NS
Sleep latency, min	25.74 ± 17.33	29.76 ± 41.54	NS
Sleep stage 1, % of TST	23.47 ± 8.46	26.63 ± 9.84	NS
Sleep stage 2, % of TST	52.15 ± 8.67	47.55 ±14.61	NS
Sleep stage 3, % of TST	4.70 ± 6.70	8.58 ± 11.55	NS
Sleep REM, % of TST	19.68 ± 9.84	17.24 ± 11.08	NS
REM latency, min	123.09 ± 81.62	141.72 ± 103.93	NS
Awakenings, total	18.09 ± 9.27	23.69 ± 8.24	NS
Awakenings index/h	4.01 ± 1.96	5.83 ± 3.75	NS
PLM index	64 ± 55	61 ± 48	NS
PLMS index	72 ± 65	66 ± 63	NS
PLMW index	38 ± 33	48 ± 37	NS
RLS-like, *n* (%)	3 (30.77)	8 (27.27)	NS

TST: total sleep time. Sleep efficiency was calculated as % of sleep during time in bed; sleep stages were calculated as % of TST; index of periodic leg movements (PLM) was calculated per hour of time in bed (PLM index), per hour of sleep (PLMS index), and per hour of wakefulness (PLMW index), awakening index = total number of awakenings in TST, RLS-like: restless legs symptoms, MSA-C: multiple system atrophy cerebellar predominant, MSA-P: multiple system atrophy parkinsonian predominant, NS: nonsignificant (*P* value > 0.05).

**Table 3:** The percentage of  “any” EMG activity in MSA patients with RBD.

	“Any” EMG activity	
	Mean ± SD (%)	
MSA-C patients	With RBD	Without RBD
	46.79 ± 21.17	7.35 ± 7.94
MSA-P patients	With RBD	Without RBD
	51 ± 36.96	17.72 ± 31.51

SD: standard deviation, RBD: REM Sleep Behavior Disorder, MSA-C: multiple system atrophy cerebellar predominant, MSA-P: multiple system atrophy parkinsonian predominant.

**Table 4:** Respiratory parameters at PSG in MSA patients.

	MSA-C (*n* = 11)	MSA-P (*n* = 26)	Significance *P* value
Sleep apnea, *n* (%)	7 (64)	11 (42)	NS
Min SaO2, %	82.22 ± 13.24	81.69 ± 10.99	NS
Average SaO2, %	94.68 ± 1.69	93.79 ± 2.80	NS

NS: not significant (*P* value > 0.05), SaO2: oxygen saturation, MSA-C: multiple system atrophy cerebellar predominant, MSA-P: multiple system atrophy parkinsonian predominant, PSG: polysomnography.
